# A genome-wide association study identifies a novel East Asian–specific locus for dementia with Lewy bodies in Japanese subjects

**DOI:** 10.1186/s10020-025-01115-7

**Published:** 2025-03-06

**Authors:** Risa Mitsumori, Yuya Asanomi, Takashi Morizono, Daichi Shigemizu, Shumpei Niida, Kouichi Ozaki

**Affiliations:** 1 Medical Genome Center, National Center for Geriatrics and Gerontology, Research Institute, 7-430 Morioka-Cho, Obu, Aichi 474-8511 Japan; 2 Department of Cardiovascular Medicine, Hiroshima University Graduate School of Biomedical and Health Sciences, 1-2-3 Kasumi, Minami-Ku, Hiroshima, 734-8553 Japan; 3 National Center for Geriatrics and Gerontology, Research Institute, 7-430 Morioka-Cho, Obu, Aichi 474-8511 Japan; 4 RIKEN Center for Integrative Medical Sciences, 1-7-22 Suehiro-Cho, Tsurumi-Ku, Yokohama, Kanagawa 230-0045 Japan

**Keywords:** Dementia with Lewy bodies, Expression quantitative trait locus analysis, Genome-Wide association study, Transcriptome-Wide association analysis, Trans-Ethnic meta-Analysis

## Abstract

**Background:**

Dementia with Lewy bodies (DLB) is the second most common type of degenerative dementia in older patients. As with other multifactorial diseases, the pathogenesis results from interactions of environmental and genetic factors. The genetic basis of DLB is not yet fully understood. Recent genomic analyses of DLB in Caucasian cohorts identified genetic susceptibility loci for DLB, but the comprehensive genomic analysis in Asians was still not performed.

**Methods:**

We conducted a genome-wide association study (GWAS) in Japanese subjects (211 DLB cases and 6113 controls) to clarify the genetic architecture of DLB pathogenesis.

**Results:**

We identified the East Asian–specific *DHTKD1* locus (rs138587229) on chromosome 10 with genome-wide significance (GWS; *P* = 3.27 $\times$ 10^–8^) and the *ICOS*/*PARD3B* locus on chromosome 2 with suggestive significance (*P* = 3.95 $\times$ 10^–7^) as novel DLB genetic risk loci. We also confirmed the *APOE* locus (rs429358, *P* < 5.0 × 10^–8^), a known risk locus for DLB and Alzheimer’s disease in Caucasians. The *DHTKD1* locus was associated with the gene expression of *SEC61A2* and showed a causal relationship with cholinesterase levels. In a trans-ethnic meta-analysis that included Japanese, UK Biobank, and other Caucasian GWAS, we confirmed the risk for DLB at *APOE* and *SNCA* loci with GWS. Transcriptome-wide association analysis identified *ZNF155* and *ZNF284* in the brain cortex and *GPRIN3* in the substantia nigra as putative causal genes for DLB.

**Conclusions:**

This is the first GWAS for DLB in East Asians, and our findings provide new biological and clinical insights into the pathogenesis of DLB.

**Supplementary Information:**

The online version contains supplementary material available at 10.1186/s10020-025-01115-7.

## Background

Dementia with Lewy bodies (DLB) is the second most common subtype of clinically heterogeneous neurodegenerative diseases in humans, after Alzheimer’s disease (AD) (Walker et al. 2015; Mueller et al. 2017). The current therapy is limited to symptomatic and supportive care. The hallmarks of DLB are neuronal α-synuclein inclusions (Lewy bodies and Lewy neurites) that are deposited in the brain cortex and limbic system and induce neuronal loss (Parkkinen et al. 2008). The pathological changes are shared with Parkinson’s disease (PD).

DLB, PD/PD dementia (PDD), and AD share the underlying genetic architecture (Meeus et al. 2012). Mutations in *SNCA* and *LRRK2* have been reported as strong risk factors for sporadic PD/DLB (Hyun et al. 2013). Mutations in *GBA1* have also been reported to be significant risk factors for DLB in a multicenter study (Nalls et al. 2013). Recent large-scale genetic analyses such as genome-wide association study (GWAS) and whole-genome sequencing analysis identified the *APOE*, *BIN1*, and *TREM175* loci in addition to the *GBA1* and *SNCA* loci for DLB susceptibility (Bras et al. 2014; Bracaglia., et al. 2018; Chia et al. 2021). *APOE* and *BIN1* are strong genetic susceptibility loci for AD (Lambert 2014); in particular, the ε4 allele of *APOE* is the main genetic risk factor. The *APOE* ε4 carriers have higher risk of AD pathology, accelerated age-dependent cognitive decline, and worse memory performance than do noncarriers (Liu et al. 2013). The *APOE* ε4 allele frequency was significantly higher in pure DLB (31.9%) and PDD (19.1%) patients than in control subjects (Tsuang et al. 2013). The *TREM175* locus has been identified in PDD-GWAS (Pankratz et al. 2009). Several genetic risk factors for DLB have been identified, mostly by large-scale analysis of subjects of Caucasian descent. However, no reports are available on DLB genetic risk factors in Asians.

In this study, we aimed to identify the genetic architecture of DLB using GWAS in the Japanese population, followed by a trans-ethnic meta-analysis with datasets from the UK Biobank and another Caucasian cohort (Chia et al. 2021). We conducted the expression quantitative trait locus (eQTL) analysis and transcriptome-wide association analysis, and investigated the possible mechanisms of DLB pathogenesis by functional annotation of trans-ethnic meta-analysis of GWAS. We also assessed the causal relationship between DLB variants and clinical data by Mendelian randomization analysis, and the genetic correlations for DLB and different diseases and traits. Our findings suggest novel genetic pathways implicated in DLB and provide further biological and clinical insights to investigate its pathogenesis.

## Methods

### Subjects and DNA samples

All 6,324 genomic DNA samples and the associated clinical data were obtained from the National Center for Geriatrics and Gerontology (NCGG) Biobank, Japan. Of these samples, 211 were from patients with DLB and 6,113 were from cognitively normal elder control subjects, all Japanese. All of the diagnoses were based on the criteria of the fourth report of the DLB Consortium (McKeith et al. 2017) by neurologists, psychiatrists, geriatricians, or a neurosurgeon, all specialists in dementia who are familiar with its diagnostic criteria. In cases with an uncertain diagnosis, the diagnosis was determined at a case conference attended by neurologists, psychiatrists, geriatricians, a neurosurgeon, neuropsychologists, and neuroradiologists. We have included patients diagnosed with possible or probable DLB in the analysis. The control subjects were cognitively normal older adults from the NCGG hospital and a cohort study of older adults in the NCGG neighborhood. Their cognitive function was confirmed by a Mini-Mental State Examination score ≥ 24 (mean: 27.7 ± 1.89) (Folstein et al. 1975). Among DLB patients, the average age was 79.1 years (56–94 years) and 63.0% were female. Among the control subjects, the average age was 72.3 years (60–93 years) and 55.8% were female. All subjects provided written informed consent. This study was approved by the ethics committee of the NCGG and conducted in accordance with the Declaration of Helsinki. Genomic DNA was extracted from peripheral blood leukocytes by standard protocols using a Maxwell RSC Instrument and a Maxwell RSC Buffy Coat DNA Kit (Promega, Madison, WI, USA).

### Genotyping and quality control for GWAS

Genome-wide genotyping of the Japanese subjects was performed by using the Infinium Asian Screening Array (Illumina, San Diego, CA, USA). We performed SNP imputation using minimac4 with the Japanese reference panel, constructed in the previous study (Koike et al. 2023), using the 1000 Genomes Project Phase 3 data (1KGP 3 [May 2013 n = 2504]) and 3,256 Japanese whole-genome sequence data from the BioBank Japan (BBJ) database. We used variants with an INFO score $\ge$ 0.3 in the association analysis. We first applied quality control (QC) filters to the subjects using PLINK 1.9 (Purcell et al. 2007): (1) sex inconsistencies (–check-sex), (2) kinship coefficient (–genome 0.3), (3) genotype missingness (–mind 0.05), and (4) exclusion of outliers from the clusters of East Asian populations in a principal component analysis that was conducted together with 1000 Genomes Phase 3 data. We next applied QC filters to the variants: (1) genotyping efficiency or call rate (–geno 0.02), (2) minor allele frequency (–maf 0.01), and (3) Hardy–Weinberg equilibrium (–hwe 0.001).

### Japanese GWAS

Logistic regression analysis adjusted for sex and age was performed using PLINK 1.9 (–logistic, –covar) (Purcell et al. 2007). Heritability, genetic correlation, and linkage disequilibrium (LD)-score regression were evaluated using LDSC (v1.0.1) (Bulik-Sullivan et al. 2015a, b; Finucane et al. 2015). All variants were annotated using ANNOVAR (avsnp150) (Wang et al. 2010). Regional association plots were generated using LocusZoom (http://locuszoom.org). To check for the secondary association signals, we conducted conditional analyses for the loci of interest by performing logistic regression on each lead variant. To check the consistency of imputed genotypes for the lead variants of interest, we used multiplex PCR-invader assay (Third Wave Technologies, Madison, WI, USA) (Ohnishi et al. 2001) by using a QuantStudio 7 Flex Real-Time PCR System (Thermo Fisher Scientific, Waltham, MA, USA).

### Genetic heritability

To estimate SNP heritability in Japanese subjects, we performed LD score regression (Bulik-Sullivan et al. 2015b; Finucane et al. 2015). We used LD scores from East Asian populations in the 1000 Genomes dataset suitable for general LD score analyses (LD score regression intercept, heritability) (Bulik-Sullivan et al. 2015b).

### eQTL analyses

SNP genotype data, which was also determined using the Infinium Asian Screening Array, and blood-based RNA sequencing (RNA-Seq) data for 1,723 Japanese subjects were obtained from the NCGG Biobank. Gene expression was quantified as transcripts per million (TPM). Each eQTL was assessed with a linear regression model using gene expression data and SNP genotype; the model was adjusted for sex and age with PLINK software (–liner) (Purcell et al. 2007).

### Two-stage least-squares Mendelian randomization (2SLS MR)

All clinical data of 513 subjects were obtained from the NCGG biobank and included nine categories: (1) anthropometry (body mass index [BMI]), (2) blood components (leukocyte count, platelet count, erythrocyte count, mean corpuscular hemoglobin, hematocrit volume, hemoglobin volume, mean cell hemoglobin concentration, mean corpuscular volume, mean platelet volume, monocyte%, lymphocyte%, neutrophil%, neutrophil count, basophil%, and eosinophil%), (3) blood glucose levels (hemoglobin A1c [HbA1c] and glucose), (4) dyslipidemia (triglycerides and total cholesterol), (5) kidney function (urea nitrogen and creatine), (6) liver function (alanine aminotransferase [ALT], aspartate transaminase [AST], gamma-glutamyl transpeptidase [γ-GT], albumin/globulin ratio, alkaline phosphatase [ALP], total bilirubin [T-BIL], indirect bilirubin [I-BIL], direct bilirubin [D-BIL], albumin [ALB], total protein, and cholinesterase [ChE]), (7) markers of inflammation (C-reactive protein and creatine kinase), (8) markers of thyroid function (thyroid-stimulating hormone [TSH], free triiodothyronine [FT3], and free thyroxine [FT4]), and (9) serum electrolytes (K, Na, Cl, Ca, and inorganic phosphorus). We performed 2SLS MR analysis (Burgess et al. 2017; Smith and Hemani 2014) using DLB-GWAS variants as genetic instrumental variables, DLB phenotype as exposure, and clinical data as outcome.

### Genetic correlation

To analyze genetic correlation between DLB-GWAS and Japanese GWAS for different phenotypes, we used LDSC (Bulik-Sullivan et al. 2015a, b). We obtained summary statistics of Japanese GWAS data from the BBJ database (PheWeb.jp) (Sakaue et al. 2021; Shirai et al. 2022). We analyzed correlations for BMI, cerebral aneurysm, depression, epilepsy, intracerebral hemorrhage, ischemic stroke, myocardial infarction, type 2 diabetes, and autoimmune disease. All GWAS summary statistics data were formatted for LD score regression filtering to HapMap3 SNPs. We used LD score files for East Asian produced by LDSC.

### Trans-ethnic meta-analysis

We used summary statistics of our Japanese GWAS, the UK Biobank genotyping data, and another European DLB-GWAS (Chia et al. 2021). To obtain summary statistics from the UK Biobank (www.ukbiobank.ac.uk/), we retrieved genotyping and clinical data from its participants. We selected 215 DLB cases according to the following criteria: (1) the subjects with ICD10 codes of DLB (G318V, V10S and F028), (2) there is no ICD10 code indicates other dementias, and (3) whose parents have no history of AD or PD in their medical history. The average age was 62.6 years (41–70 years) and 45.1% were female. We used 169,279 subjects according to the following criteria for control: no cognitive disorder, 60 years of age or older, and no parents affected by dementia. The average age was 64.1 years (60–73 years) and 52.2% were female. We applied QC filters to the SNP and Indel markers: (1) genotyping efficiency or call rate (–geno 0.95), (2) minor allele frequency (–freq 0.01), and (3) Hardy–Weinberg equilibrium (–hwe 0.001). To obtain summary statistics of the UK Biobank GWAS, we used logistic regression adjusted for sex and age in PLINK software (–logistic) (Purcell et al. 2007). After QC, we finally obtained 4,712,774 variants.

Summary statistics of another European DLB-GWAS were obtained from NHGRI-EBI (https://www.ebi.ac.uk/gwas/publications/33589841) (Chia et al. 2021). We converted the genome coordinates of all variants from GRCh38 to hg19 with LiftOver software (http://genome.ucsc.edu/cgi-bin/hgLiftOver) and obtained 7,630,112 variants.

Trans-ethnic meta-analysis of the summary statistics from Japanese GWAS, UK Biobank GWAS, and the other European GWAS was performed using METASOFT (v2.0.0) (Han and Eskin 2011). Calculation of the Meta-P values was based on the Han and Eskin’s modified random-effects model. A total of 1,850,275 variants with common-effect alleles and the same directionality of beta value between the three cohorts were selected for association analysis. We considered SNPs with *P*_RE_ < 1 $\times$ 10^–6^ to be suggestive significance.

### Gene-based association analysis

We used the trans-ethnic meta-GWAS data and FUMA (https://fuma.ctglab.nl) (Watanabe et al. 2017) to analyze 18,132 genes, and applied Bonferroni correction to account for multiple testing (significance threshold: *P* = 2.8 $\times$ 10^–6^). Variants were annotated using the HapMap 3 reference panel of East Asian. Summary statistics of gene-based analysis was obtained by using gene expression data at GTEx v8 using FUMA.

### Protein–protein interaction (PPI) network analysis

We performed the PPI network analysis based on five significant genes (false discovery rate *P*-value:* P*_FDR_ < 0.05) identified in the gene-based analysis using NetworkAnalyst web application (Zhou et al. 2019). The PPI network was visualized using Cytoscape (v3.7.2) (Otasek et al. 2019).

### Transcriptome-wide association study (TWAS)

To estimate the association between predicted gene expression levels and GWAS summary statistics, we conducted a TWAS using MetaXcan (Barbeira et al. 2018). We used precomputed prediction models of gene expression in brain tissues (amygdala, anterior, caudate, cerebellar, cerebellum, cortex, frontal, hippocampus, hypothalamus, nucleus, putamen, spinal cord, and substantia) with the LD reference data in the GTEx v7 expression model from PredictDB (Gamazon et al. 2015) and the summary statistics of trans-ethnic meta-analysis. We set the Bonferroni significance level taking into account the number of genes available for each tissue.

## Results

### DLB-GWAS in the Japanese population

**Fig. 1:**
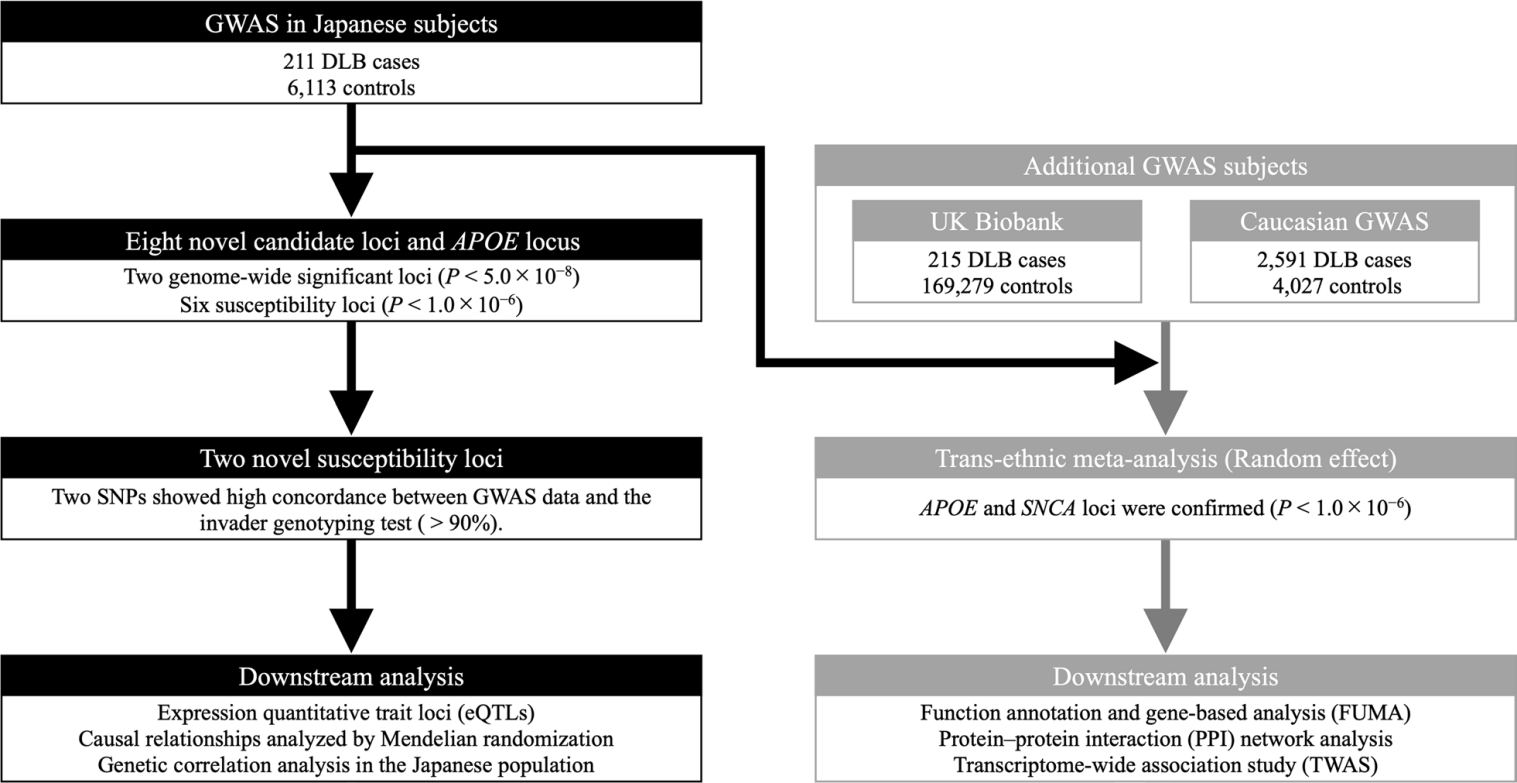
The workflow of this study. Black background, ethnicity-specific analyses; gray background, trans-ethnic analyses

**Fig. 2:**
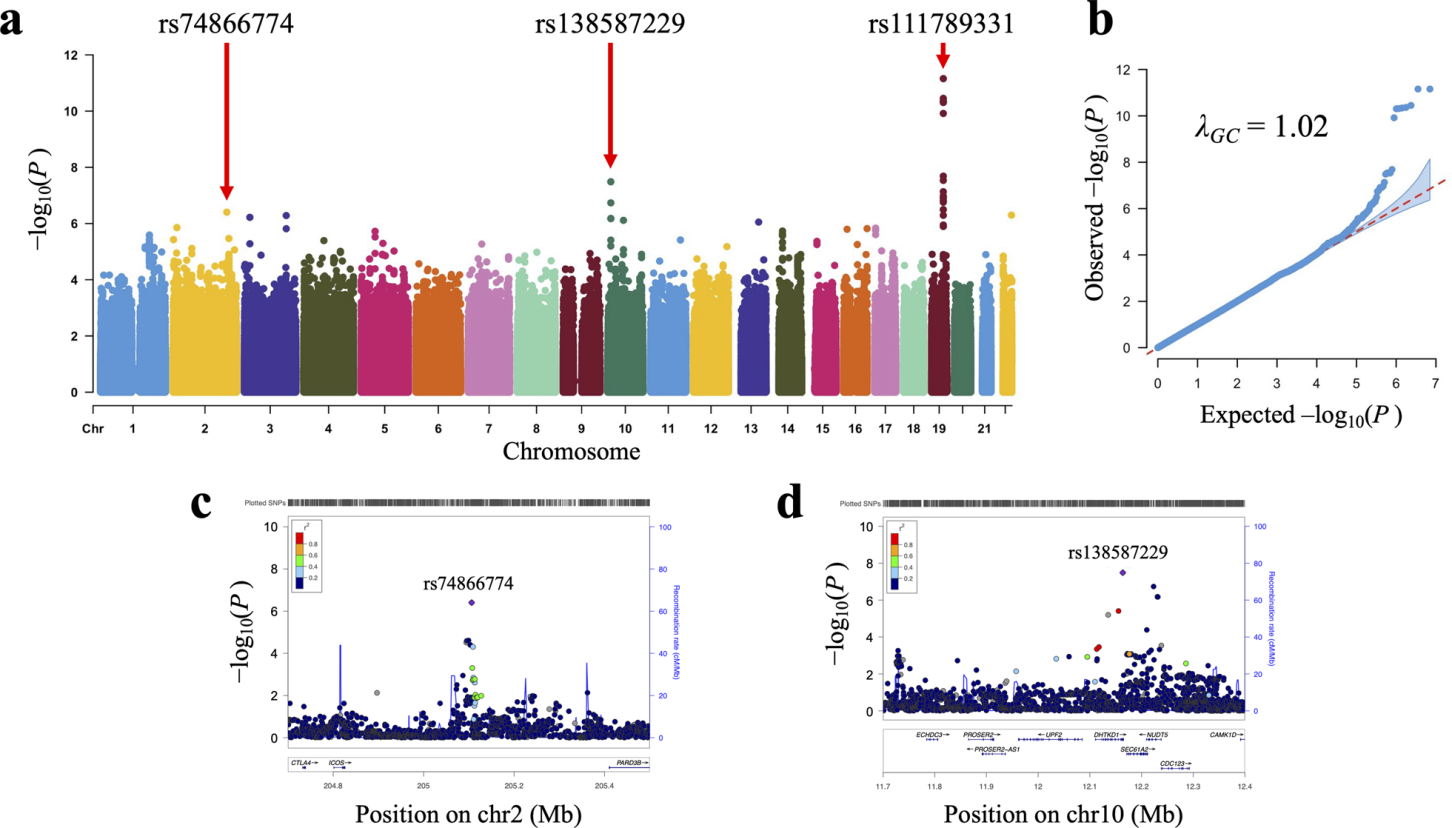
GWAS for DLB in the Japanese population. **a** A Manhattan plot of the Japanese DLB-GWAS. **b**) Quantile–quantile plot of the GWAS *P*-values. **c**, **d** Regional association plots for (**c**) the new susceptibility locus on chromosome 2 and (**d**) the new genome-wide significant locus on chromosome 10

The workflow of this study is shown in Fig. 1. We conducted a DLB-GWAS in Japanese subjects (211 cases and 6,113 controls) from the NCGG Biobank (Fig. 2 and Table 1). We used 8,040,962 variants in the autosomes that passed QC filters. The genomic inflation factor (λ_GC_) was 1.02 (Fig. 2b); the LD-score regression indicated that the inflation was primarily due to polygenic effects (LD-score regression intercept = 1.00). The estimated SNP heritability (*h*^*2*^) was 23.5% (standard error of the mean = 12.0%). The DLB-GWAS identified two GWS loci (*P* < 5.0 $\times$ 10^–8^) and six candidate susceptibility loci (*P* < 1.0 $\times$ 10^–6^) (Supplementary Fig. S1 and Supplementary Table S1). Although two SNPs had suggestive *P*-values (*P* = 8.88 $\times$ 10^–7^, chromosome13; *P* = 5.02 $\times$ 10^–7^, chromosome 22; Supplementary Fig. S1d, e), they had no neighboring SNPs with linkage disequilibrium; we considered them to have likely resulted from imputation errors and excluded them from further analysis. As the lead variants of five of the six remaining candidate loci were determined by imputation, we examined the accuracy of the genotyping data for these five loci with the PCR-invader assay and a subset of the DNA samples used in GWAS, and evaluated the concordance rate (Number of mismatching genotypes/Number of subjects) of the imputed variants (Supplementary Table S1). Three lead variants showed low concordance (rs117975786, 5.3%; rs190336540, 73.5%; rs147178120, 73.1%) and were excluded from further analysis.

**Table 1:** Lead variants of two GWS loci (*P* < 5.0 × 10^–8^) and a suggestive locus (*P* < 1.0 × 10^–6^) identified in Japanese DLB-GWAS

Chr	Position (hg19)	dbSNP ID	A1	A2	OR	95% CI	*P*	Minor allele frequency								Annotated gene †	Closest TSS gene	Location
								Japanese GWAS			gnomAD							
								All	Case	Control	All	European (Finnish)	African	American	East Asian			
2	205106145	rs74866774	T	C	2.37	1.70–3.32	3.95 × 10^–7^	0.06	0.11	0.06	0.08	0.08	0.09	0.03	0.06	*NRP2*	*PARD3B*	Intergenic
10	12163771	rs138587229	C	G	3.72	2.34–5.93	3.27 × 10^–8^	0.02	0.06	0.02	0.0007	0	0	0	0.02	*dhtkd1*	*SEC61A2*	3′ UTR of *DHTKD1*
19	45427125	rs111789331	A	T	2.59	1.97–3.40	6.98 × 10^–12^	0.1	0.19	0.1	0.12	0.25	0.03	0.09	0.1	*APOE*	*APOC1P1*	Intergenic

^†^Genes were annotated by using Open Targets (https://www.opentargets.org)

*Chr* chromosome, *A1* minor allele, *A2* major allele, *OR* odds ratio, *CI* confidence interval, *TSS* transcription start site

One of the two GWS loci was the *APOE* locus, a known risk locus for DLB in Caucasians (Chia et al. 2021; Shigemizu et al. 2021; Bellenguez et al. 2022), and its lead SNP was rs111789331 (*P* = 6.98 $\times$ 10^–12^; Table 1), while another well-known DLB-risk *SNCA* locus found in Caucasians (Bras et al. 2014; Bracaglia., et al. 2018; Chia et al. 2021) was not replicated in our Japanese GWAS (Supplementary Fig. S2). The other GWS locus was novel, with the lead SNP rs138587229 (*P* = 3.27 $\times$ 10^–8^), and was located within the 3′-UTR of *DHTKD1*. The rs138587229 SNP seemed to be East Asian–specific, as suggested by the absence of its frequency data for other ethnic groups in the gnomAD database (Table 1). Locus rs74866774 with suggestive significance was located between the *ICOS* and *PARD3B* genes on chromosome 2. To verify the secondary association signals at these three DLB-associated loci, we used the lead variants rs111789331, rs138587229, and rs74866774 in conditional analyses. No secondary independent association signals were detected in any of these loci (Supplementary Fig. S3).

### eQTL analyses

The lead SNP of the novel GWS locus rs138587229 was located within the 3′-UTR of *DHTKD1*, and had *NUDT5*, *SEC61A2* and *CDC123* neighboring on chromosome 10. The data from eQTL analyses of rs138587229 and the neighboring genes using blood RNA-Seq data of 1,723 Japanese subjects registered with the NCGG Biobank are shown in Table 2. *DHTKD1* (the closest gene), *NUDT5*, and *CDC123* had no statistical significance. The second closest gene, *SEC61A2*, a nominal eQTL (*P* = 0.02), showed increased gene expression. No eQTL was found in the *ICOS* /*PARD3B* suggestive locus for rs74866774 or its proxy variants.

**Table 2:** eQTL analysis for the lead variant of the novel GWS locus using blood RNA-Seq

Gene	rs138587229	
	*P*_eQTL_ without Age/Sex	*P*_eQTL_ with Age/Sex
*NUDT5*	0.98	0.93
*SEC61A2*	**0.03**	**0.02**
*DHTKD1*	0.08	0.64
*CDC123*	0.51	0.47

Bold indicates statistical significance

### Causal relationships between DLB-risk loci and clinical data

**Fig. 3:**
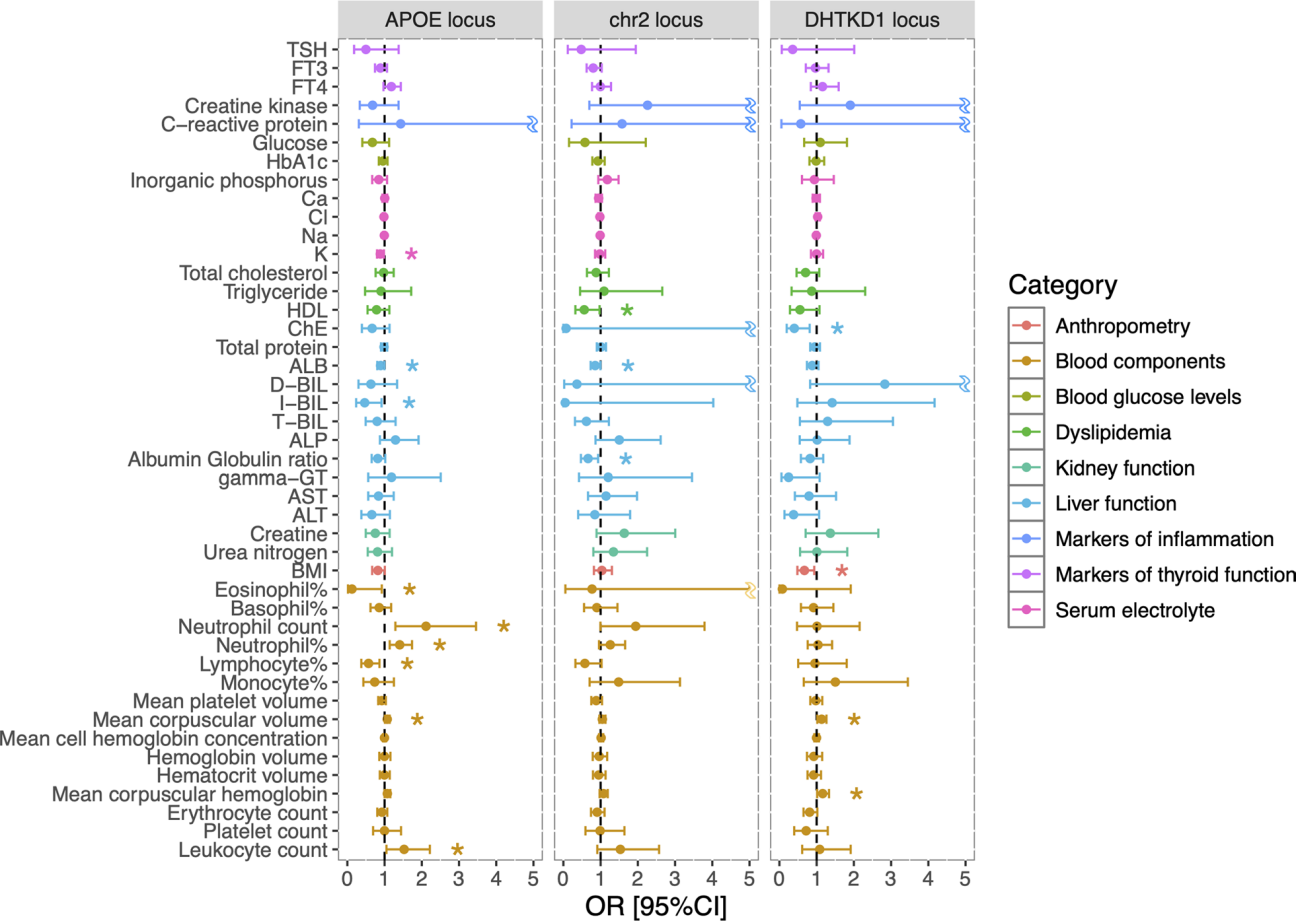
Causal relationship between three DLB variants and clinical data. Causal effects are shown for rs111789331 in the *APOE* locus, rs74866774 on chromosome 2, and rs138587229 in the *DHTKD1* locus. Each dot represents the odds ratio (OR) with an error bar indicating the 95% confidence interval (CI). Asterisks indicate significant relationships (*P* < 0.05)

In 2SLS MR analyses, we estimated the causal effects of DLB variants (Fig. 3). A list of 44 clinical indicators for 513 subjects used in the analyses is shown in Supplementary Table S2. Causal effects were identified for rs74866774 in the *ICOS* /*PARD3B* locus on chromosome 2 for albumin/globulin ratio (OR [95% CI] = 0.66 [0.47–0.93]), ALB (0.85 [0.73–1.00]), and high-density lipoprotein (0.56 [0.32–0.97]); for rs138587229 in the *DHTKD1* locus for BMI (0.67 [0.48–0.94]), ChE (0.40 [0.20–0.81]), mean corpuscular hemoglobin (1.16 [1.01–1.33]), and mean corpuscular volume (1.13 [1.02–1.26]); for rs111789331 in the *APOE* locus for potassium (0.88 [0.80–0.98]), ALB (0.89 [0.80–1.00]), I-BIL (0.47 [0.24–0.92]), leukocyte count (1.53 [1.05–2.22]), mean corpuscular volume (1.07 [1.01–1.14]), lymphocyte% (0.57 [0.38–0.86]), neutrophil% (1.41 [1.14–1.74]), neutrophil count (2.11 [1.29–3.46]), and eosinophil% (0.12 [0.02–0.93]).

### Genetic correlations across different diseases and traits

We investigated the genetic overlap between our GWAS data and phenotypes from other GWAS by using GWAS summary statistics from the BBJ database (Sakaue et al. 2021; Shirai et al. 2022). Among the nine phenotypes analyzed (BMI, cerebral aneurysm, depression, epilepsy, intracerebral hemorrhage, ischemic stroke, myocardial infarction, type 2 diabetes, and autoimmune disease; Supplementary Fig. S4), we found a significant negative correlation for ischemic stroke (*P* = 0.035).

### Trans-ethnic meta-analysis

To improve the statistical power to detect further DLB susceptibility loci, we performed a trans-ethnic meta-analysis using a combination of summary statistics from our Japanese GWAS, UK Biobank data, and European GWAS data of NHGRI-EBI (Chia et al. 2021). A total of 1,850,275 variants were used for this analysis with a random-effect model by METASOFT (Han and Eskin 2011). This analysis confirmed two loci located in the known DLB-risk genes, *APOE* and *SNCA* (*P* < 5 $.0\times$ 10^–8^, Supplementary Fig. S5). We also conducted a gene-based analysis (Supplementary Fig. S6 and Supplementary Table S3). *APOE*, *APOC1*, and *TOMM40* were identified as significantly associated genes (*P*_Bon_ < 0.05), whereas *SNCA* and *PVRL2* were detected with suggestive significance (*P*_FDR_ < 0.05). For these five genes, we performed a PPI network analysis and identified three hub molecules, APOE, TOMM40, and SNCA, in the network for DLB pathogenesis (Supplementary Fig. S7). In the analysis of expression in 11 brain tissues, amygdala and putamen basal ganglia reached Bonferroni-corrected significance (*P*_Bon_ = 0.044 and *P*_Bon_ = 0.033, respectively) for DLB pathogenesis (Supplementary Table S4).

### Brain tissue–specific TWAS

To investigate candidate causal genes associated with DLB, we used the results of trans-ethnic meta-analysis GWAS and conducted TWAS in 13 brain tissues (Table 3). In the brain cortex, *ZNF155* and *ZNF284*, near the *APOE* locus in chromosome 19, reached Bonferroni-corrected significance (*P*_Bon_ = 0.040 and *P*_Bon_ = 1.0 $\times$ 10^–4^, respectively). In the substantia nigra, the intronic variant of *SNCA* on chromosome 4, rs1471484, was significantly associated with *GPRIN3* (*P*_Bon_ = 8.1 $\times$ 10^–5^).

**Table 3:** Novel causal genes identified in the brain tissue–specific TWAS

Brain tissue	Chr	Position	Ensembl gene ID	Gene symbol	Z score	Effect size	*P*
Cortex	19	44472014	ENSG00000204920	*ZNF155*	4.4	4.8	9.31 × 10^–6^
Cortex	19	44576297	ENSG00000186026	*ZNF284*	− 5.6	− 30.8	2.44 × 10^–8^
Substantia nigra	4	90157537	ENSG00000185477	*GPRIN3*	5.5	2.7	4.05 × 10^–8^

*Chr* Chromosome

## Discussion

Our Japanese DLB-GWAS identified one novel GWS locus on chromosome 10 and one novel suggestive locus on chromosome 2, and also confirmed the known *APOE* locus on chromosome 19. While the lead variant of the newly identified GWS locus, rs138587229, was located within the 3′-UTR of *DHTKD1* (Fig. 2d), eQTL analysis revealed that the locus associated with the increased gene expression of the neighboring *SEC61A2* (Table 2). However, the present study has some limitations. The sample size is relatively small, and the case–control ratio is not balanced, which could cause some bias. The statistical power was insufficient to detect the variants with a lower odds ratio (< 1.5) in the sample size of our population. Thus, the analyses with additional sample sizes and replication analyses with the other Asian cohorts may provide further insights into the genetic architecture of DLB.

*SEC61A2* encodes the SEC61 α2 subunit, which is a component of SEC61 channel-forming translocon complex in the endoplasmic reticulum (ER) membrane (Daniel et al. 2008; Connerly et al. 2005). *SEC61A2* shares 95% sequence identity with *SEC61A1*, whereas almost all published data on Sec61α were obtained for *SEC61A1* (Lang et al. 2012; Zhou and Schekman 1999; Zehner et al. 2015). The translocon recognizes nascent polypeptides through signal recognition particles and imports them from the cytosol into the ER (Daniel et al. 2008; Linxweiler et al. 2017). The translocon also exports secretory proteins from the ER to the cytosol for degradation (Lang et al. 2012; Zhou and Schekman 1999; Zehner et al. 2015). The loss-of-function experiments with the *Drosophila* ortholog of Sec61α (DSec61α) have revealed that Dsec61α accelerates the accumulation of abnormal proteins and neuron death by facilitating the export of unfolded proteins from the ER to the cytosol in expanded polyglutamine–mediated neuronal toxicity (Kanuka et al. 2003). Conversely, hypoactive Dsec61α inhibits the export of unfolded proteins from the ER to the cytosol and protects against neuronal toxicity. Sec61 translocon is involved in the activation of the unfolded protein response (UPR) under ER stress through the Sec61–IRE1α complex formation (Sundaram et al. 2017). In several neurodegenerative diseases, including DLB and PD, ER stress and the accumulation of misfolded proteins have been demonstrated, and ER stress activates the UPR (Baek et al. 2016; Costa et al. 2020; Guan et al. 2021; Kim et al. 2022; Ren et al. 2021). Understanding the detailed molecular mechanisms of ER stress and how it is related to neurodegeneration may clarify the DLB pathogenesis and lead to the development of effective therapies.

The lead SNP of GWS locus, rs138587229, showed a causal relationship with cholinesterase levels (Fig. 3). In the cortex of patients with DLB and PDD, the levels of acetylcholine are strongly decreased, and are even lower than in patients with AD pathology (Rolinski et al. 2012_)._ Cholinesterase inhibitors have been widely used to improve cognitive function in patients with DLB, PDD, and AD (Hershey and Coleman-Jackson 2019; Tan 2014). Although the relationship between blood cholinesterase levels used in this study and those brain cholinesterases should be carefully discussed, it has been reported that plasma AChE activity positively correlated with brain β-amyloid levels determined by PIB-PET examination (Alkalay, et al. 2013). We also indicated causal effects for rs138587229 on BMI, MCH, and MCV. BMI levels have been reported to show a decrease in disease progression in patients diagnosed with DLB (Borda et al. 2022). In contrast, there was no reports for association of MCH or MCV with DLB so far. The GWS locus on chromosome 10 identified in this study may contribute to DLB risk by influencing cholinesterase levels, potentially through mechanisms involving enhanced *SEC61A2* expression.

A comparison between our DLB-GWAS data and the summary statistics of the other Japanese GWAS identified a negative correlation for ischemic stroke (Supplementary Fig. S4). This suggests that the genetic risk factors associated with DLB could decrease the risk of ischemic stroke, and vice versa. A postmortem study has shown that the frequency of cerebrovascular lesions of various intensities was lower in DLB patients than in both PD patients and controls, and that ischemic strokes and hemorrhages were not observed in DLB (Jellinger 2003). However, cohort studies in Sweden and Taiwan reported a higher risk of ischemic stroke in DLB patients than in AD patients and controls (Cheng et al. 2018; Fereshtehnejad et al. 2014), but our results support the opposite trend. Further research is needed to resolve this discrepancy.

In TWAS using the summary statistics of trans-ethnic meta-analysis, we identified an association between DLB and *GPRIN3* in the substantia nigra (Table 3). *GPRIN3* encodes a member of the GPRIN (G-protein-regulated inducer of neurite outgrowth) family and is highly expressed in the striatum, where it mediates the function of the dopamine receptor D2, playing a major role in striatal physiology (Karadurmus et al. 2019). Being a partner of β-arrestin-2, GPRIN3 regulates dopamine receptor desensitization and affects selective behavioral tasks (Mototani et al. 2018). *GPRIN3*-knockout mice have reduced locomotor activity, increased anxiety-like behavior in the elevated maze test, and reduced locomotor response to dopamine stimulation (Mototani et al. 2018). Severe nigrostriatal degeneration and consequent dopamine transporter loss occur in DLB (Piggott et al. 1999). Our trans-ethnic meta-analysis is consistent with these DLB pathologies. On the other hand, *ZNF155* and *ZNF284*, also identified in TWAS, have no reports indicating the potential significance or association with DLB. Further investigations are desired to elucidate the associations between these genes and DLB.

## Conclusions

Our first DLB-GWAS in the Japanese population has revealed a novel genetic architecture of DLB; we anticipate that the findings presented here will provide novel biological and clinical insights into the pathogenesis of DLB.

## Supplementary Information

Additional file 1: Table S1. Candidate DLB susceptibility loci identified in Japanese GWAS

Additional file 2: Table S2. Clinical data used for 2SLS MR analyses

Additional file 3: Table S3. Candidate genes identified in gene-based association analysis

Additional file 4: Table S4. Summary statistics of gene-based tissue-expression analyses for 11 brain tissues

Additional file 5: Fig. S1. Regional association plots for the susceptibility loci other than those shown in Figure 2. Fig. S2. Regional association plot of *SNCA* locus in Japanese DLB-GWAS. Fig. S3. Conditional analysis for loci identified in Japanese DLB-GWAS. Fig. S4. Genetic correlation analyses. Fig. S5. Trans-ethnic meta-analysis of DLB. Fig. S6. Gene-based association analysis using trans-ethnic meta-GWAS data. Fig. S7**.** PPI network analysis based on the 5 candidate genes identified in gene-based association analysis.

## Data Availability

No datasets were generated or analysed during the current study.

## Abbreviations

AD: Alzheimer’s disease
BBJ: BioBank Japan
DLB: Dementia with Lewy bodies
ER: Endoplasmic reticulum
eQTL: Expression quantitative trait locus
GWAS: Genome-wide association study
GWS: Genome-wide significance
LD: Linkage disequilibrium
NCGG: National center for geriatrics and gerontology
PD: Parkinson’s disease
PDD: PD dementia
PPI: Protein–protein interaction
RNA-Seq: RNA-sequencing
UPR: Unfolded protein response
