# Supplementary material for: A genome-wide association study identifies a novel East Asian–specific locus for dementia with Lewy bodies in Japanese subjects
Source: Mol Med. 2025 Mar 6;31:87. doi: 10.1186/s10020-025-01115-7 (PMC11884146; doi:10.1186/s10020-025-01115-7)
Supplement: Supplementary file 5 — Additional file 5: Fig. S1. Regional association plots for the susceptibility loci other than those shown in Figure 2. Fig. S2. Regional association plot of SNCA locus in Japanese DLB-GWAS. Fig. S3. Conditional analysis for loci identified in Japanese DLB-GWAS. Fig. S4. Genetic correlation analyses. Fig. S5. Trans-ethnic meta-analysis of DLB. Fig. S6. Gene-based association analysis using trans-ethnic meta-GWAS data. Fig. S7. PPI network analysis based on the 5 candidate genes identified in gene-based association analysis. [file 10020_2025_1115_MOESM5_ESM.pdf]

Fig. S1. Regional association plots for the susceptibility loci other than those shown in Figure 2.

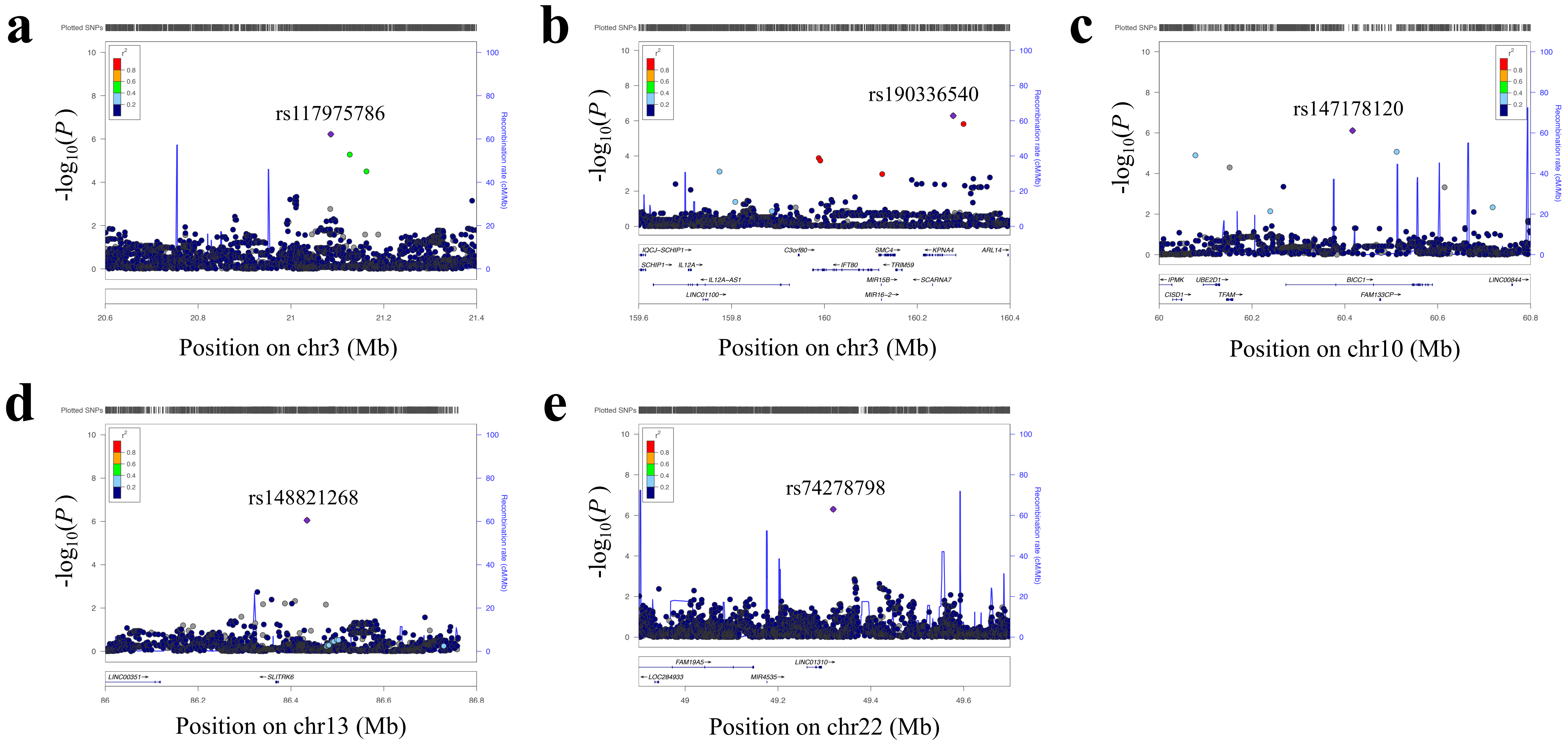

Lead variants: **a**) rs117975786, **b**) rs190336540, **c**) rs147178120, **d**) rs148821268, and **e**) rs74278798.

Fig. S2. Regional association plot of *SNCA* locus in Japanese DLB-GWAS.

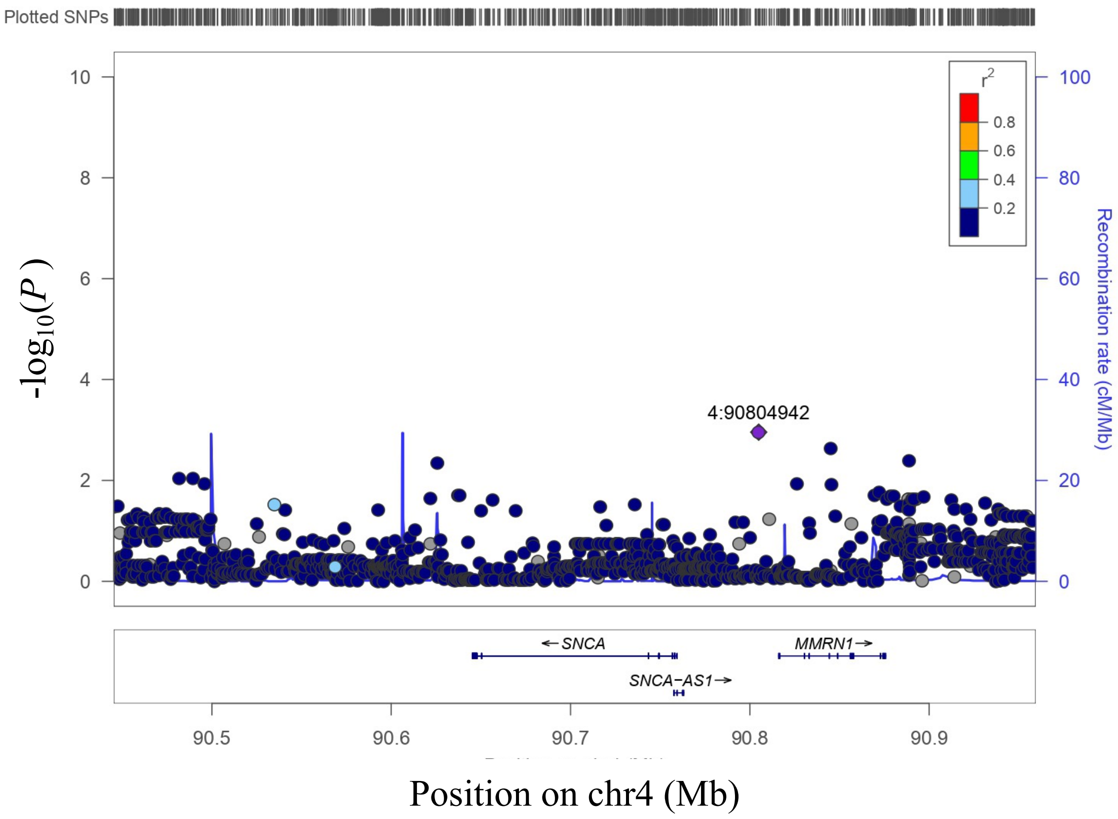

Regional association plot of well-known DLB-risk *SNCA* locus on chromosome 4 reported in Caucasian GWAS.

Fig. S3. Conditional analysis for loci identified in Japanese DLB-GWAS.

a **b** **c**

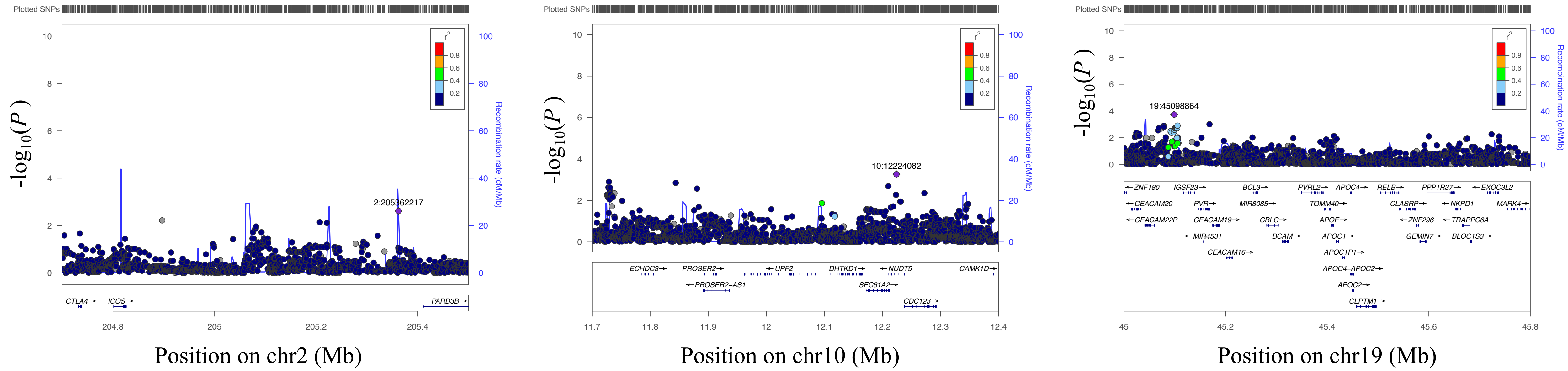

Regional association plots of **a**) rs74866774 on chromosome 2, **b**) rs138587229 on chromosome 10, and **c**) rs111789331 on chromosome 19.

Fig. S4. Genetic correlation analyses.

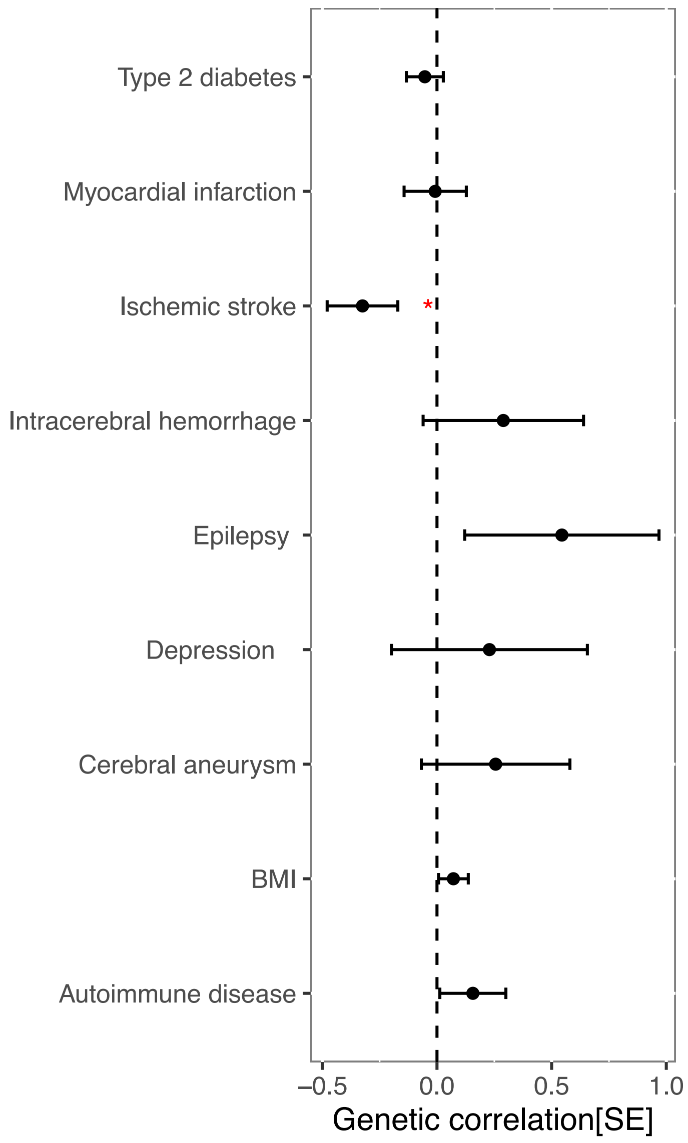

Genetic correlations between DLB and nine phenotypes are shown. Asterisk indicates significant correlation ($P < 0.05$). Error bars indicate standard error (SE).

Fig. S5. Trans-ethnic meta-analysis of DLB.

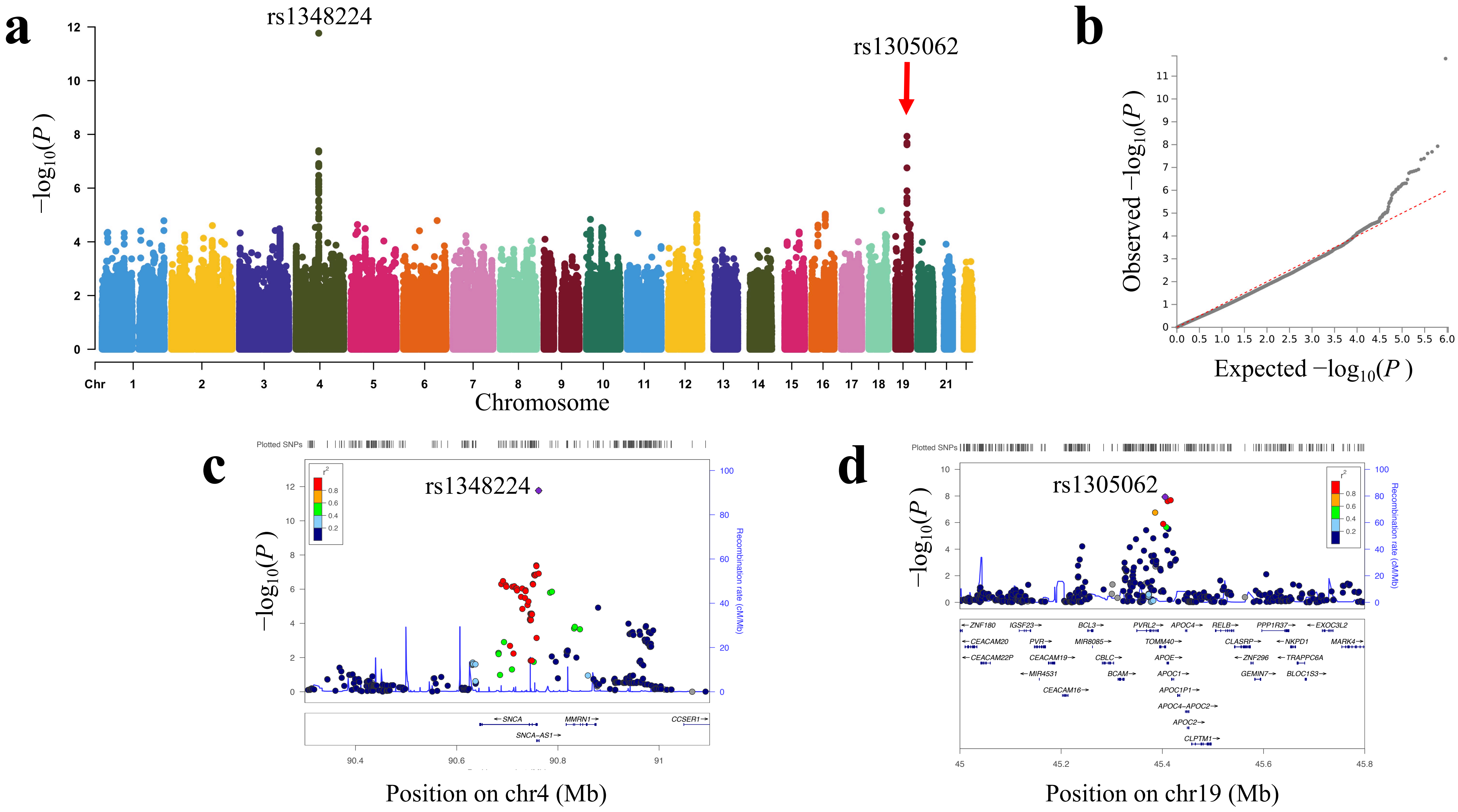

a) Manhattan plot. **b**) Quantile–quantile plot. **c,d**) Regional plots of (**c**) the *SNCA* locus and (**d**) the *APOE* locus.

Fig. S6. Gene-based association analysis using trans-ethnic meta-GWAS data.

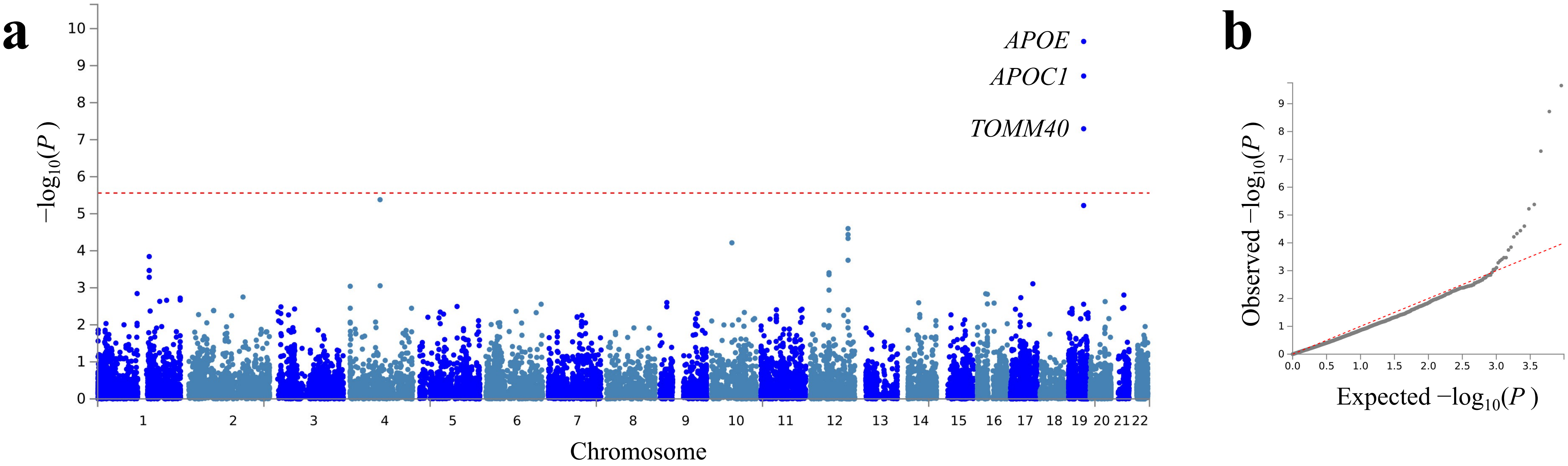

a) Manhattan plot. Red line indicates the Bonferroni-corrected *P*-value threshold. **b**) Quantile–quantile plot.

Fig. S7. PPI network analysis based on the 5 candidate genes identified in gene-based association analysis.

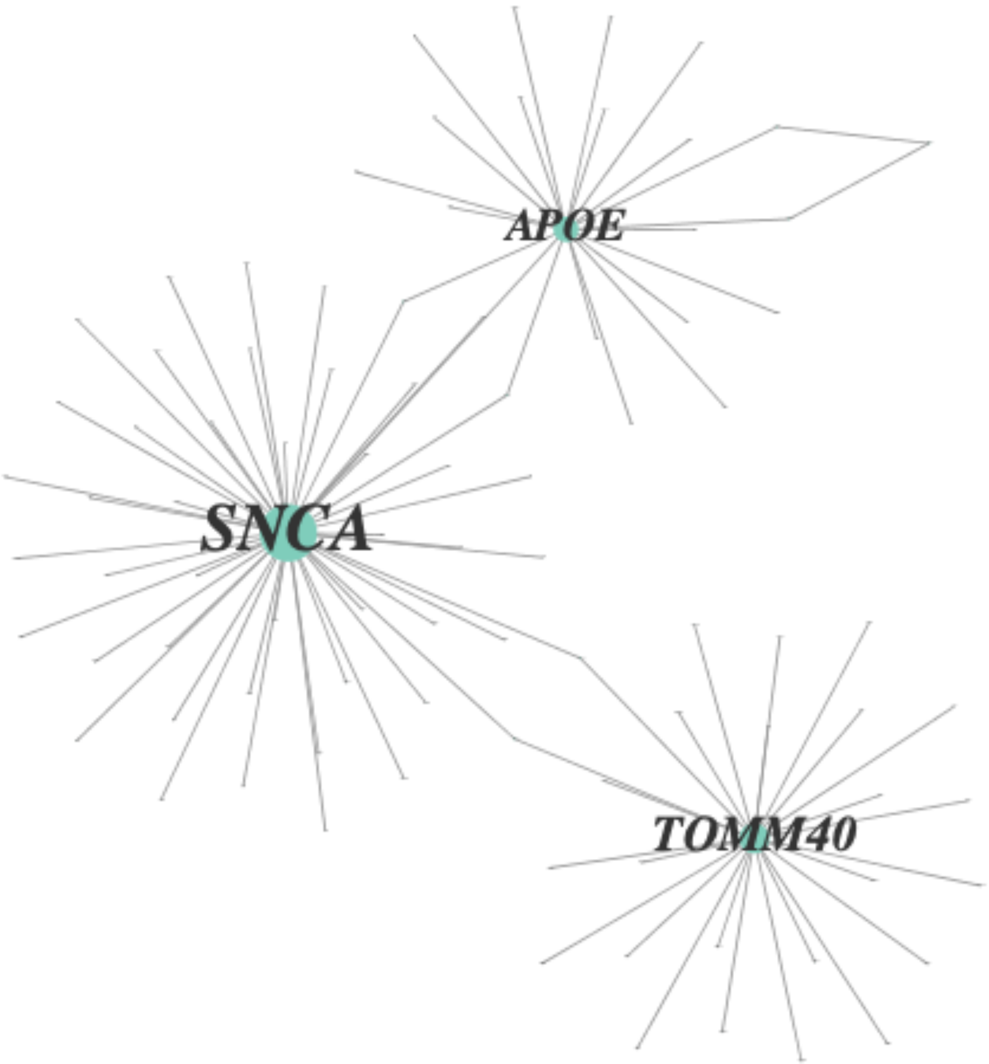

PPI network was visualized in Cytoscape. Gene symbols are displayed for nodes with degree of centrality > 20.
